# Measuring and Predicting Service Providers’ Use of an Evidence-Based Parenting Program

**DOI:** 10.1007/s10488-019-00934-1

**Published:** 2019-03-30

**Authors:** Émilie Charest, Marie-Hélène Gagné

**Affiliations:** École de Psychologie, Université Laval, 2325 rue des Bibliothèques, Pavillon F.-A.-Savard, 11e étage, Québec, QC G1V 0A6 Canada

**Keywords:** Implementation science, Triple P—Positive Parenting Program, Service providers, EBP usage, Multidimensional index

## Abstract

This study addressed the predictors of service providers’ use of a multi-level evidence-based program (EBP). Of the 92 trained providers participating in the study, 67 (72.8%) used the EBP at least once. A multidimensional index of the amount of usage (MUI) was created using three indicators. Providers’ self-efficacy and the amount of training they had received predicted their amount of usage. The community to which the providers belonged was also associated with their amount of usage. The findings underline the importance of studying many indicators of usage in implementation research and considering both provider-level and broader contextual variables as determinants of the use of EBPs.

Analyses of the implementation of evidence-based parenting and family support programs are essential to ensure that interventions are used consistently and that children and families derive reliable benefits from them (Durlak and DuPre 2008). Understanding how these evidence-based programs (EBPs) are successfully implemented requires the assessment of various implementation factors, including their acceptability, adoption, appropriateness, feasibility, fidelity, implementation cost, penetration, and sustainability (Proctor et al. 2011). Adoption (i.e., intention to try an EBP or actions taken to this end), penetration (i.e., its integration into a service setting) and sustainability (i.e., the extent to which it is maintained within a service setting) refer to its usage by trained providers. This study focuses on provider’s use of an EBP.

Follow-up studies of trained providers (e.g., Sanders et al. 2009; Seng et al. 2006) have generally shown that approximately one third of providers fail to use a given EBP after training. Among the remaining two-thirds, who report using it, the extent to which they use it remains unclear. Non-usage and low usage have many consequences, including a hidden cost for the agencies that pay to train providers who subsequently do not deliver the EBP as expected (Asgary-Eden and Lee 2012), as well as poorer reach and lower coverage (Shapiro et al. 2012). The current literature suggests that the use of an EBP may be influenced by factors at multiple levels (Aarons et al. 2011). These include characteristics of the providers (e.g., attitudes and self-efficacy), the EBP (e.g., amount and quality of training provided, perceived cost-effectiveness of the EBP), and the organizational context (e.g., financial and human resources, access to supervision and support). Neighborhood effect, defined as the community influence on individual, social or economic outcomes, could also apply to implementation factors such as usage (Bobba and Gignoux 2014; Rauktis et al. 2010).

While the theoretical literature points to a wide range of factors likely to have an impact on providers’ use of an EBP, fewer empirical studies have used these factors to predict usage. Among these, Finley et al. (2015) conducted a national survey of providers within the U.S. Department of Veterans Affairs PTSD clinical teams to identify individual and organizational factors associated with the uptake of two EBPs. The results suggest that provider attitudes and organizational factors such as staffing and work relationships had an important impact on treatment selection and the quality of care provided. Although the results of this study are interesting, they do not provide information on the implementation context, the training received by providers, or the time that had elapsed between training and data collection.

Moreover, these results are hardly generalizable to multi-level EBPs, for which usage is much more complex to assess. Multi-level programs include several levels of intervention of increasing intensity, each catering to a different level of need or dysfunction. Available empirical evidence suggests that these programs are promising at the population level, as they maximize the efficiency of interventions, reduce the costs involved, and serve a greater proportion of the community (Asgary-Eden and Lee 2012; Gagné et al. 2012; Nelson and Caplan 2014). Given that multi-level EBPs have only recently begun to be developed and evaluated, very few empirical studies have examined the factors predicting their use. However, due to the growing popularity of “proportionate universalism” in public health (Marmot 2010), which refers to delivering universal services at a scale and intensity proportionate to the degree of need, multi-level EBPs are expected to develop and expand in the near future. Thus, interest in multi-level EBPs is increasingly relevant in implementation research (Poissant 2014).

The present study examines the implementation and sustainability of the Triple P—Positive Parenting Program (Sanders 1999) during the first 2 years of its implementation in the province of Quebec, Canada. Triple P is a multi-level EBP aimed at parents of children from birth to age 12, and seeks to improve parenting competence and confidence by modifying dysfunctional parenting practices and interaction patterns that contribute to children’s behavioral problems. This EBP includes 5 levels of intervention, the first being a social marketing campaign used to promote positive parenting in the population. The present study examines Levels 2 to 5. Level 2 includes two modalities: public seminars aimed at all parents in the community, and brief one-on-one consultations to support parents who have a few concerns regarding their child’s behavior. Level 3 is for parents dealing with a specific problem, and includes approximately four individual consultations. Level 4 is aimed at parents of children with severe behavioral problems, and includes five group sessions supported by three individual phone-counseling sessions. Level 5 is offered in either a group or individual modality (approximately five sessions), and includes intensive support for families facing serious problems. Meta-analyses report that Triple P has shown positive effects on various parenting and child outcomes including parents’ well-being, parental stress, children’s behavioral and emotional problems, and the parent–child relationship (De Graaf et al. 2008; Nowak and Heinrichs 2008; Sanders et al. 2014; Thomas and Zimmer-Gembeck 2007).

Recent studies have examined providers’ use of Triple P through surveys (Turner et al. 2011) or interviews (Sanders et al. 2009; Seng et al. 2006; Shapiro et al. 2012) conducted among services providers, 6 onths (Sanders et al. 2009; Seng et al. 2006; Turner et al. 2011) or 2 years (Shapiro et al. 2012) after their initial training. These studies assessed usage by asking providers whether or not they had used Triple P at any time since their initial training (Sanders et al. 2009; Seng et al. 2006; Shapiro et al. 2012), whether they had used it with a given minimum number of families (Shapiro et al. 2015), and/or the number of families with which they had used it (Shapiro et al. 2012; Turner et al. 2011). These studies revealed that between 15% and 38% of providers who had completed training and accreditation processes (i.e., passed the competency evaluation) did not subsequently use Triple P. They also showed that several provider- and organization-level characteristics significantly predicted usage, including the extent of training received (Sanders et al. 2009; Seng et al. 2006), the availability of post-training support from the organization, professional relationships and support from peers or colleagues in the workplace (Sanders et al. 2009), fit of the EBP with ongoing duties, the perceived benefit of the intervention for children and families (Sanders et al. 2009; Shapiro et al. 2012), and provider self-efficacy (Sanders et al. 2009; Shapiro et al. 2012; Turner et al. 2011). Regarding training, Sanders et al. (2009) found that providers who became high users of Triple P tended to be trained in Group Triple P (Level 4). Turner et al. (2011) also found that intervention supports (quality of format and materials) and barriers (management difficulties and lack of fit) predicted usage through their impact on practitioner self-efficacy. Shapiro et al. (2012) concluded that any examination of the factors impacting implementation must be accompanied by efforts to define and measure usage.

These studies show important limitations. The collected data involved retrospective estimates of usage, which could be vulnerable to recall bias. Also, there is a lack of consensus regarding the definition and measurement of usage across studies. Most authors have conceptualized usage as a dichotomous variable (e.g. Sanders et al. 2009; Seng et al. 2006; Shapiro et al. 2015). However, it is critical to determine whether, in addition to merely providing an EBP, service providers are doing so to a reasonable extent, which some authors refer to as the “amount of usage” (Shapiro et al. 2012). In studies examining usage as a continuous variable, this variable has generally been assessed using only one indicator. The most frequent indicator reported is the number of families with which the providers used the EBP. However, this indicator is largely affected by the modality of the intervention used by the provider: in the case of a multi-level EBP such as Triple P, it may underestimate usage when providers mainly conduct individual interventions, and overestimate it when providers conduct group interventions or public seminars.

Several authors have pointed to the need to measure multiple aspects of EBP delivery in order to achieve a comprehensive picture of implementation processes (Hasson 2010). The creation of a multidimensional usage index would thus make it possible to capture many aspects of usage, reflecting the different conceptualizations of this construct and allowing for a more accurate and exhaustive assessment of the extent of the dissemination of the EBP. Such an index would be particularly relevant in the case of a multi-level EBP such as Triple P. Because providers can use different intervention modalities (individual interventions, group interventions, and/or public seminars) and, with multi-level training, more than one level/modality, any indicator of usage is hardly comparable from one provider to another, making usage very complex to assess using a single indicator.

## Current Study

The present study examined the predictors of providers’ use of Triple P during the first two years of its active implementation. The first goal was thus to examine the predictors of usage versus non-usage. Providers were considered users if they used Triple P with families at least once. The predictors examined included the provider’s level of education, profession, experience in the family-child care field, experience in the organization, attitudes toward EBPs, self-efficacy, perceived organizational barriers and facilitators to implementation, amount of training, and accreditation. The second goal was to develop a multidimensional usage index (MUI) based on prospective measures: the number of Triple P activities conducted, the number of parents reached, and the total duration of the interventions conducted during the first 2 years of active implementation. The third goal was to examine the predictors of the amount of usage as indicated by the MUI. The predictors examined were the same as for usage versus non-usage.

## Method

In January 2015, Triple P was implemented for the first time in the province of Quebec, Canada. This initiative was carried out as a demonstration project through a community-university partnership devoted to the prevention of child maltreatment. All five levels of Triple P were implemented in two health care catchment areas (Community 1 and Community 2) by a number of partners working in collaboration (child welfare services, primary social care services, community organizations, primary schools and child daycare centers). These two communities were chosen because of the large population of families with children from birth to age 12 living within their territories (between 7700 and 10,000) and the vulnerability of their populations, whether in terms of poverty or child maltreatment rates (with between 6% and 22% of 0–12 year-olds living under the low-income threshold, and between 14.1‰ and 14.3‰ of 0–12 year-olds being investigated following a report of child maltreatment made to child protection services). Given the possible neighborhood effects on the implementation factors assessed, community was used as a control variable in the analyses.

### Participants

Ninety-six service providers from 21 organizations received training from *Triple P International* (TPI) during the fall of 2014, and 19 of them received additional training during the fall of 2015. Of these 96 trained providers, 92 participated in the study (response rate of 95.8%). Participants’ characteristics are presented in Table 1. As expected in this domain of practice, the majority of providers were women. Over 94% of providers held a post-secondary diploma or degree. One third of providers had a social work background, and two-thirds worked for government health care agencies. Fifty-five providers belonged to Community 1 and 37 belonged to Community 2. Thirty-one providers (33.7%) attended only one level of training, 44 (47.8%) attended two levels, and 17 (18.5%) attended three or four levels. Seventy-six providers (82.6%) achieved accreditation for at least one level of training. The rate of providers who achieved accreditation was higher in Community 2 (94.6%) than in Community 1 (74.5%), χ^2^ = 6.189, dl = 1, *p* = 0.013, ϕ = 0.259.

**Table 1:** Description of the Sample (N = 92)

	*n* (valid %)
Health care catchment area	
Community 1	55 (59.8)
Community 2	37 (40.2)
Gender	
Female	85 (92.4)
Male	7 (7.6)
Highest level of education completed	
High school diploma or less	5 (5.6)
Technical/academic junior college diploma	27 (30.0)
Undergraduate degree	45 (50.0)
Graduate degree	13 (14.4)
Profession	
Social work	32 (36.0)
Special education or psychoeducation	23 (25.0)
Early childhood education	10 (11.2)
Nursing	8 (9.0)
Other	16 (17.4)
Type of organization	
Government health care agency	62 (68.1)
Community organization (non-profit)	22 (24.2)
Public primary school	4 (4.4)
Subsidized child daycare center	3 (3.3)

	*M (SD)*
Years of experience in this organization	10.45 (8.59)
Years of experience in the family-child care field	13.29 (9.57)

*Note* Valid percentages exclude missing data

### Measures

The data came from three sources: a pre-implementation survey, registration forms and attendance lists for the training sessions, and ongoing implementation monitoring instruments completed during the first two years of the implementation.

#### Pre-implementation Survey

This survey was filled out by service providers prior to their first Triple P training session in fall 2014. This questionnaire collected information on the participants’ characteristics: gender, level of education, profession, community, type of organization, experience in this organization, and experience in the family-child care field. Among these, four variables were examined as predictors of usage. *Highest level of education completed* was dichotomized: 0 = technical/academic junior college diploma or less, 1 = university degree (undergraduate or graduate). *Profession* was broken down into four categories: 1 = Social work, 2 = Special education, early childhood or psychoeducation, 3 = Nursing, and 4 = Other (including psychology, elementary school education, communication, youth correctional intervention, and other). *Number of years of experience in the family*-*child care field* and *number of years of experience in this organization* were measured as continuous variables. *Community* was used as a control variable in the analyses: 0 = Community 1, 1 = Community 2.

The pre-implementation survey also assessed the providers’ attitudes and perceptions toward the implementation of Triple P through four standardized instruments: the Evidence-Based Practice Attitude Scale (EBPAS; Aarons 2004), the Parent Consultation Skills Checklist (PCSC; Turner and Sanders 1996), the Organizational Readiness for Change measure (ORC; Lehman et al. 2002), and the Factors Related to Program Implementation measure (FRPI; Mihalic and Irwin 2003).

##### Attitudes Toward EBPs

Providers’ attitudes toward EBPs were assessed using the four subscales of the EBPAS (Aarons 2004): *Appeal* (intuitive appeal of EBPs; 4 items, e.g., “If you received training in an intervention that was new to you, how likely would you be to adopt it if it was intuitively appealing?”), *Requirements* (likelihood of adopting EBPs given requirements to do so; 3 items, e.g., “If you received training in an intervention that was new to you, how likely would you be to adopt it if it was required by your supervisor?”), *Openness* (openness to new practices; 4 items, e.g., “I like to use new types of interventions to help my clients”), and *Divergence* (perceived divergence between research-based interventions and the provider’s current practice; 4 items, e.g., “I know better than academic researchers how to care for my clients”). Items were scored on a 5-point scale (1 = not at all; 5 = to a very great extent). Aarons et al. (2007) reported adequate internal consistency for each of the four subscales (Cronbach’s alphas ranged from 0.66 to 0.93). In the present study, the EBPAS demonstrated good internal consistency for each subscale (α = 0.72, 0.93, 0.87 and 0.73, respectively).

##### Self-efficacy in Parent Consultations

Providers’ self-efficacy or confidence in conducting behavioral family interventions with parents was assessed using the PCSC (Turner and Sanders 1996). This questionnaire was adapted for each level of Triple P, with the number and content of the items being tailored to the specific skills or competencies required to provide that particular level. This checklist contained between 17 and 20 items measuring perceived proficiency in core skills, including assessment, active parenting skills training, dealing with process issues, and clinical application of positive parenting strategies. The items were rated on a 7-point scale (1 = not at all confident; 7 = very confident) and the total score was obtained by calculating a mean score out of 7. Providers were asked to complete this measure before each training session they attended. As our variable of interest was the providers’ pre-training level of confidence, only the first measure completed was used in the current study. The PCSC showed high internal consistency, with α coefficients ranging from 0.94 to 0.96.

##### Perceived Training Needs

Providers’ perception of their training needs related to working with parents was assessed using the *Training needs* subscale of the ORC (Lehman et al. 2002). Seven items (e.g., “You feel immediate needs to get specialized training for assessing patient problems and needs”) were scored on a 5-point scale (1 = strongly disagree; 5 = strongly agree). In Lehman et al.’s validation study, this subscale showed good psychometric properties, including good discriminant validity, good construct validity and acceptable internal consistency, with a Cronbach’s α coefficient of 0.57. In the present study, the subscale demonstrated good internal consistency (α = 0.87).

##### Perceived Adequacy of the Organization’s Physical Environment

Providers’ perception of the adequacy of their organization’s offices and physical space available to support the implementation of Triple P was assessed using the *Offices* subscale of the ORC (Lehman et al. 2002). Four items (e.g., “Offices here allow the privacy needed for individual counseling”) were scored on a 5-point scale (1 = strongly disagree; 5 = strongly agree). In Lehman et al.’s validation study, this subscale showed good psychometric properties, with a Cronbach’s α coefficient of 0.62. In the present study, it also demonstrated good internal consistency (α = 0.79).

##### Perceived Organizational Facilitators and Barriers to Implementation

Providers’ perception of the organizational facilitators and barriers to the implementation of Triple P was assessed using three subscales from the FRPI (Mihalic and Irwin 2003): *Ideal Agency characteristics* (13 items), *Ideal Staff characteristics* (7 items), and *Ideal Champion characteristics* (4 items). The *Ideal Champion characteristics* scale was adapted slightly to fit the implementation context: the items remained the same but the term *Team leader* (referring to the providers’ immediate supervisor) was used instead of *Champion*. Supervisors played an important role in the adoption and implementation of Triple P. They had a close relationship with service providers and motivated, encouraged and guided them through the implementation process. The items were scored on a 5-point scale (1 = Significant barrier; 5 = Significant facilitator) and measured, from the providers’ perspective, the extent to which these characteristics represented anticipated facilitators or barriers to the implementation of Triple P. Sample items include: “Throughout the process of implementing Triple P, how much do you think each of the following factors will be an asset or a barrier: Open lines of communication between agency officials, program staff, and service providers; Service provider’s motivation; and Supervisors’ skills and knowledge. Mihalic and Irwin reported good internal consistency for each of the three subscales (α = 0.84, 0.88, and 0.81, respectively). In our study, the three subscales also showed good internal consistency (α = 0.93, 0.91, and 0.95, respectively).

#### Training and Accreditation

Registration forms and attendance lists for training sessions were used to document the training and accreditation processes completed by providers. Two measures were derived from the collected information: amount of training and accreditation. The *amount of training* corresponded to the number of levels of training the provider attended in 2014 and 2015 (when applicable). This variable was dichotomized: 0 = only one level of training, 1 = 2 or more levels of training. *Accreditation* corresponded to whether or not the provider achieved accreditation for at least one level of training. This variable was dichotomized: 0 = did not achieve any accreditation, 1 = achieved accreditation for at least one level of training.

#### Usage

Drawing on the literature in the field, a computerized application was developed to monitor various implementation parameters. This application contained monitoring instruments—tailored to each level of Triple P (2–5)—that providers completed for each Triple P activity and session conducted. An activity was defined as the series of sessions/consultations conducted with a family as part of a Triple P intervention. For example, for Primary Care Triple P (Level 3), a series of 4 individual consultations was considered as one activity. For Seminars and Brief Triple P (Level 2), one public seminar was considered as one activity. Among other aspects, these tools provided information on the number of activities conducted by each provider over a 2-year period, the duration of each session, and the number of parents reached by the intervention. For Level 2, providers indicated the date of the activity, the duration, and the number of parents present. For Levels 3–5, providers indicated, for each session of the activity, the date, the duration, and the presence/absence of each enrolled parent.

*Usage* was assessed using one dichotomous indicator: whether or not the provider used Triple P at least once during the 2-year period following their initial training (0 = non-user; 1 = user). The *amount of usage* was measured using three continuous indicators: the number of Triple P activities conducted, the number of parents with whom the provider had used Triple P, and the total duration of the interventions conducted (in hours). The scores for these three indicators were calculated by summing up the data for all the levels of Triple P implemented by the provider (Levels 2 to 5).

### Procedures

Recruiting providers to participate in the study was the responsibility of the managers of the agencies involved in the initiative. All providers on their teams of staff were encouraged to receive Triple P training, with the exception of providers who planned to leave the organization before the end of the two-year demonstration project. Providers were asked to sign a commitment form explaining what was expected of them. They had to be willing to fill out questionnaires, give their opinion on Triple P and monitor their Triple P interventions. They were thus informed, prior to training, that their participation in the training involved their participation in the research. All procedures were approved on July 14, 2014 by the Ethical Review Board (ERB) of the Centre jeunesse de Québec—Institut universitaire (University-based youth center), through the Multi-Centre Research Ethics Review Mechanism of the Quebec Ministry of Health and Social Services (MSSS), approval number: MP-QJC-IU-13-017.

In fall 2014, approximately 2 weeks prior to training, providers were invited to complete the pre-implementation survey. An envelope containing an information sheet, the survey, and a return envelope was mailed or handed out to them. Providers were authorized by their supervisor to complete the survey during their working hours. Completing and returning the survey was considered consent to participate in the study.

From January 2015 to December 2016 (24 months), providers used Triple P with parents of children from birth to age 12 seeking help for which they considered it appropriate. Providers were asked to monitor their Triple P activities using the computerized application described above. Providers could access this application using electronic tablets provided by the research team during the 2-year period following initial training. This application made it possible for the data to be directly transmitted to the research team via a secure computer server. Extended training and support were offered by the research team to help providers use the application. Paper versions were used by a few providers who were reticent to use this technology, and as backups in case of digital failure.

Providers were asked to contact the research team each time they started a new intervention. Once all the sessions of this intervention were completed, providers were asked to submit the data online or return the completed paper form to the research team. The completion and transmission of the data were closely and continuously monitored by the research team to ensure the validity of the collected data and minimize missing data. Periodical follow-up calls were made by the research team to the providers to ensure that the latter completed and submitted the data shortly after the intervention was completed.

### Strategy for Analysis

#### Interdependency of the Data Sampling

Because providers came from different organizations embedded within the two communities, interdependencies in the data collected from providers housed within the same type of organization or the same community were assessed. There were no significant correlations among the responses of individuals within organizations, or within communities, for any predictor or outcome variable. The intraclass correlation coefficients (ICC) ranged from 0 to 0.13, which, according to Koo and Li’s (2016) guidelines, are indicative of poor reliability. The assumption of independence of observations was thus respected, supporting a global analysis of the data.

#### Predictors of Usage

Data were first screened for outliers and to assess normality and multicollinearity. Exploratory analyses of the data were conducted, and correlations between usage and the following predictors were examined: highest level of education completed, profession, number of years of experience in the family-child care field, number of years of experience within this organization, amount of training, accreditation, attitudes toward EBPs, self-efficacy in parent consultations, perceived training needs, perceived adequacy of the organization’s physical environment, and perceived organizational facilitators and barriers to implementation. Any predictors that were significantly associated with usage were entered into a logistic regression (“enter” method) to predict usage versus non-usage. Community was entered as a control variable in the logistic regression.

#### The MUI and Predictors of the Amount of Usage

In creating the MUI, descriptive data relating to the providers’ use of Triple P during the 2-year demonstration project were explored and summarized. Due to the presence of some extreme data, the three continuous indicators of the amount of usage (the number of Triple P activities conducted, the number of parents reached, and the total duration of the interventions conducted) were winsorized (97th percentile cutoff). Prior to conducting a factor analysis to combine the three indicators into an integrative measure of the amount of usage, associations between the indicators were examined using the Kaiser–Meyer–Olkin (KMO) test. The KMO test is a measure of how well suited the data are for factor analysis: it measures sampling adequacy for each variable in the model and for the complete model. The statistic is a measure of the proportion of variance among variables that might be common variance. The KMO index varies between 0 and 1, with values closer to 1 indicating better associations between variables. A score under 0.70 is considered mediocre, 0.70 and above good, and 0.80 and above excellent.

Because of the heterogeneity of the providers’ practice (use of many levels [2 to 5] and modalities of intervention: individual interventions, group interventions, and/or public seminars), it was expected that the associations between the three indicators would not be high enough to conduct a valid factor analysis. Indeed, the KMO test was mediocre (0.66), supporting the pertinence of using a more sophisticated statistical approach to ensure sampling adequacy before combining the indicators into an integrative measure of the amount of usage.

Thus, to account for the heterogeneity of the providers’ practice, the first step of the data analytic strategy was to identify homogeneous groups of users within the sample of providers who used Triple P at least once (N = 67). For this purpose, a latent class analysis (LCA) was conducted based on the levels of Triple P and the modalities of intervention the providers used during the two-year demonstration project. This analysis was conducted using MPlus software (Version 7; Muthén and Muthén 1998–2012). The appropriate number of classes was determined based on the Bayesian Information Criterion (BIC; Schwarz 1978), where smaller values represent a better fit, and the Lo-Mendall-Rubin Likelihood Difference Test (LMR; Lo et al. 2001) and Parametric Bootstrapped Likelihood Ratio Test (PBLR), which compare a model with K profiles to a model with K − 1 profiles and provide the *p* value of the likelihood difference (a significant p-value supports the retention of a more complex solution with at least K profiles). Class separation was also examined based on entropy and average posterior probability values, with values closer to 1 indicating a more accurate classification. Finally, the sample size in each class and the meaningfulness of the classes were examined.

Because the providers within each class showed similar patterns of practice (levels and modalities of intervention used during the two-year demonstration project), they were considered to be comparable in terms of the indicators of the amount of usage. To allow for comparison between classes, the second step of the data analytic strategy consisted in standardizing the indicators of the amount of usage within each previously determined class.

These standardized indicators were used to create the MUI in the third step of the data analytic strategy. Associations between the standardized indicators were examined using the KMO test to ensure adequate sampling adequacy. An exploratory factor analysis was then conducted using SPSS (Version 24) to examine the validity of the combination of the three standardized indicators in the form of an index. Once the MUI was created, correlations with the predictors of the amount of usage (i.e., the same predictors as for usage versus non-usage presented above) were examined. Predictors that were significantly associated with the MUI were entered into a linear multiple regression (using the “enter” method) to predict the amount of usage. Community was entered as a control variable in the regression.

## Results

### Descriptive Data Relating to Usage

Of the 92 providers participating in the study, 67 (72.8%) used Triple P with families at least once, while 25 (27.2%) did not use Triple P at all during the first two years of implementation. Among the providers who used Triple P at least once, approximately one-third (37.3%) used only one level of Triple P, while 26 providers (38.8%) used two different levels, and 16 providers (23.9%) used more than two levels of intervention.

### Predictors of Usage

Four predictors were significantly associated with usage: highest level of education completed (*ϕ* = − 0.36), number of years of experience in the family-child care field (ρ = 0.22), amount of training (*ϕ* = 0.36), and accreditation (*ϕ* = 0.23). Amount of training and accreditation shared a high level of common variance (*ϕ*^2^= 0.39). Thus, to avoid multicollinearity, only the amount of training, which showed the highest correlations with the usage variable, was entered into the regression model.

A logistic regression was conducted to explore the associations between the predictors and usage, controlling for the community (see Table 2). The model explained 25.4% of the variance (Cox and Snell 1989) and significantly predicted usage, *p* < 0.001. Holding a technical/academic junior college diploma or less positively predicted usage, as compared to holding an undergraduate or graduate degree (*OR *= 22.3). The amount of training also positively predicted usage (*OR *= 6.4). Neither experience in the family-child care field nor community were signficantly associated with usage.

**Table 2:** Summary of logistic regression analysis for variables predicting program use (N = 92)

Variable	*OR*	*B*	*SE*	Wald	*p*
Highest level of education completed	22.33	3.11	1.15	7.35	0.007
Experience in the family-child care field	1.05	0.05	0.04	1.44	0.230
Amount of training	6.44	1.86	0.63	8.73	0.003
Community	0.98	− 0.02	0.69	2.76	0.977

### The MUI

Within the sub-sample of providers who used Triple P at least once (N = 67), the number of Triple P activities conducted ranged between 1 and 35 (*M *= 12.9, *SD *= 10.7), the number of parents reached ranged between 1 and 275 (*M *= 89.5, *SD *= 96.9), and the total duration of interventions conducted ranged between 0.2 and 154.0 h (*M *= 43.4, *SD *= 50.8). The associations between the three indicators were moderate to high. The number of Triple P activities conducted was highly associated with the total duration of the interventions conducted (*r* = 0.74), and the number of parents reached (*r* = 0.71). The total duration of the interventions conducted was moderately associated with the number of parents reached (*r* = 0.37).

To determine the optimal number of classes of users, solutions with 1-profile through 5-profiles were estimated. LCA fit indices and sample sizes for each model are presented in Table 3. The three-class solution resulted in the best model fit (lowest BIC, highest entropy, significant LMR and PBLR tests). The three classes were distinct from one another, and class assignments were highly reliable. Class 1 providers (n = 20, 29.9%) conducted mostly Level 4 and Level 5 interventions, in either a group or individual modality, or both. They often used more than one level of intervention, and these were mainly “long-term” interventions, that is, more than 4 sessions. Among these providers, the number of Triple P activities conducted ranged between 2 and 15 (*M *= 6.7, *SD *= 4.1), the number of parents reached ranged between 8 and 78 (*M *= 35.3, *SD *= 21.6), and the total duration of the interventions conducted ranged between 11.9 and 154.0 h (*M *= 58.3, *SD *= 38.2). Class 2 providers (n = 19, 28.4%) conducted mostly Level 2 (seminars and brief consultations) and Level 3 interventions. They often used more than one level, and these were mainly one-off or “short-term” interventions, i.e., 4 sessions or less. Among these providers, the number of Triple P activities conducted ranged between 1 and 35 (*M *= 12.9, *SD *= 10.7), the number of parents reached ranged between 1 and 275 (*M *= 89.5, *SD *= 96.9), and the total duration of the interventions conducted ranged between 0.2 and 147.7 h (*M *= 43.4, *SD *= 50.8). Class 3 providers (n = 28, 41.8%) conducted Level 2, 3 or 4 interventions. The specificity of this profile is that most providers used only one level of intervention. In other words, they were less apt to adjust the intervention to the parents’ needs than providers from the other two classes. Among these providers, the number of Triple P activities conducted ranged between 1 and 35 (*M *= 5.4, *SD *= 7.2), the number of parents reached ranged between 1 and 146 (*M *= 34.5, *SD *= 38.7), and the total duration of the interventions conducted ranged between 1.25 and 153.96 h (*M *= 35.2, *SD *= 43.1).

**Table 3:** Model fit indices for one- to five-class solutions

Model	AIC	BIC	*Entropy*	LMR *p*	BLRT *p*
2 Classes	565.430	603.257	0.800	0.0003	0.0000
3 Classes	567.304	625.305	0.879	0.0138	0.0000
4 Classes	573.258	651.434	0.989	0.0152	0.3636
5 Classes	583.466	681.816	0.864	0.0840	0.6667

*AIC* Akaike Information Criteria, *BIC* Bayesian Information Criteria, *LMR p p*-value of the Lo-Mendall-Rubin Likelihood Difference Test, *BLRT p p*-value of the Bootstrapped Likelihood Ratio Test

After standardizing the indicators of the amount of usage within each class, the associations between the three indicators in the sample of providers who used Triple P at least once (N = 67) were high (*r* ranging from 0.63 to 0.85). The KMO test was good (0.70), suggesting that the sampling was adequate to conduct a valid factor analysis with the three indicators. Results of the exploratory factor analysis showed that only the first component had an eigenvalue over 1.00, suggesting that a one-factor solution best reflected the data. The one-component solution provided an accurate summary of the relationships in the data, explaining over 81% of the total variability in the data. Factor loadings indicated that the contribution of the three indicators were equivalent (0.84, 0.92, and 0.94), suggesting that a mean score would be equivalent to the factorial score. The mean score was perfectly correlated to the factorial score (*r* = 1.00), and the reliability between the three indicators was high (Cronbach’s alpha = 0.89). The MUI ranged from − 1.20 to 3.02 (*M* = 0.00, *SD* = 0.89). The distribution was slightly asymmetric (skewness = 1.18) and platykurtic (kurtosis = 1.07), but according to the guidelines for severe non-normality (i.e., skewness > 2; kurtosis > 7) proposed by Curran et al. (1996), the normality assumption was met.

### Predictors of the Amount of Usage

Five predictors were significantly associated with the amount of usage as indicated by the MUI: experience in the family-child care field (*r* = 0.29), experience within the organization (*r* = 0.35), amount of training (ρ = 0.43), accreditation (ρ = 0.25), and self-efficacy (*r* = 0.31). Some predictors shared a high level of common variance: number of years of experience in the family-child care field and number of years of experience within the organization (*r*^2^ = 0.63); amount of training and accreditation (*ϕ*^2^ = 0.39). Thus, to avoid multicollinearity, within these pairs of predictors, only that showing the highest correlation with the amount of usage was entered into the regression model (i.e., experience within the organization and amount of training).

A linear multiple regression analysis was conducted to explore the associations between experience within the organization, amount of training and self-efficacy, and the amount of usage as indicated by the MUI, controlling for the community (see Table 4). The model explained 37.1% of the variance and significantly predicted the amount of usage (*p* < 0.001). Two variables were positively and significantly associated with the amount of usage as indicated by the MUI: amount of training (*B *= 0.86, *p* < 0.001), and self-efficacy (*B *= 0.27, *p* = 0.004). Also, belonging to Community 2 was associated with a higher MUI (*B *= 0.45, *p* = 0.028). Experience within the organization was not a significant predictor (*p* = 0.163).

**Table 4:** Summary of Linear multiple regression analysis for variables predicting amount of program use as indicated by the PUI (N = 67)

Variable	*B*	*SE B*	*β*	*p*
Community	2.20	0.20	0.45	0.028
Number of years of experience within the organization	1.40	0.10	0.14	0.163
Amount of training	5.54	0.16	0.86	0.000
Self-efficacy	2.87	0.09	0.23	0.004

## Discussion

The present study examined providers’ use of Triple P during the first two years of its active implementation. The MUI was developed based on three prospective measures: the number of Triple P activities conducted, the number of parents reached, and the total duration of the interventions conducted. Predictors of usage versus non-usage, as well as the predictors of the amount of usage (as indicated by the MUI) were examined.

Seventy-three percent (73%) of providers used Triple P with families at least once during the first 2 years of active implementation. This percentage is similar to that observed in previous follow-up studies of trained providers (e.g., Sanders et al. 2009; Seng et al. 2006; Shapiro et al. 2012). Thus, it appears that increased attention to and monitoring of the amount of usage in the present initiative did not increase the proportion of users compared to previous research. This could be partly explained by the turnover rate in both communities (30.9% in Community 1 and 24.3% in Community 2). Almost half of the providers who did not use Triple P (n = 12) left their position during the first year following training (of these, six left during the first three months). This occurred in the context of a reorganization of the health and social services network, which created some instability, as people involved in the initiative changed jobs shortly following Triple P training.

One variable—the amount of training—was a significant predictor of both usage (versus non-usage) and amount of usage, suggesting its important contribution in terms of understanding providers’ use of EBPs. Regarding usage, providers who attended two or more levels of training were six times more likely to use Triple P. The amount of training was also the most important predictor of the amount of usage. This confirms the importance of properly preparing providers to implement EBPs (Glisson et al. 2008; Sanders et al. 2009). It also suggests that, in the case of a multi-level EBP such as Triple P, training across levels (i.e., receiving at least two levels of training), could be an asset for successful implementation. Being skilled in more than one level could give providers more opportunities to use the different components of the EBP. In the context of the present study design, it is also possible that providers who were initially more interested in Triple P asked to attend more levels of training. Similarly, providers who had a positive experience with Triple P during the first year of active implementation may have asked to attend additional training during the fall of 2015. Subsequently, these providers may also have been more likely to use the EBP because of their interest in it. Another possible explanation is that, given the time and energy they had invested, providers who attended two or more levels of training felt more engaged in the implementation of Triple P. Moreover, given the financial investment made by the organization and the partnership, as well as the decreased workload and leave granted to allow providers to attend the training sessions, the latter may have felt a degree of pressure—or higher expectations—from their organization and/or supervisor to use the EBP.

One predictor, the highest level of education completed, was specific to usage versus non-usage, and was, in fact, its most significant predictor: providers holding a technical/academic junior college diploma or less were 22 times more likely to use Triple P than providers holding an undergraduate or graduate degree. This result might be explained by the kind of work providers are expected to do. It is possible that, compared to providers holding a technical diploma, providers holding an undergraduate or graduate degree occupy positions involving a broader range of tasks that do not directly involve parents (e.g., management, coordination, supervision). They might spend a smaller proportion of their work time conducting consultations with parents regarding child behavior, and thus have fewer opportunities to use Triple P. This result highlights the importance of considering the job category when selecting providers for training. When implementing a parenting EBP such as Triple P, selected providers should conduct parent consultations as part of their typical workload, and be able to apply the EBP in a variety of practice contexts in order to ensure usage.

Consistent with previous studies, self-efficacy at baseline significantly predicted the amount of usage. This supports the theory that providers who are confident that they have the skills needed to deliver an EBP, even previous to specific training, are more likely to do so (Turner and Sanders 2006).

Community was also significantly associated with the amount of usage. This confirms the importance of the broader context when it comes to the implementation of EBPs. Yet, two previous studies conducted one year prior to the implementation of Triple P showed no difference between the two communities regarding community readiness to prevent child maltreatment (Gagné et al. 2018; Thomas 2018). The communities were similar with regard to various indicators: attitudes toward child maltreatment, legislation, mandates and policies, will to address the problem, material resources, and informal social resources. Regarding the characteristics of their respective populations, the two communities were similar in terms of the large population of families with children from birth to age 12 living within their territories and with regard to child maltreatment rates. It is therefore unlikely that these variables influenced the amount of usage. However, the communities differed in terms of poverty (with 21.2% of 0–12 year-olds living under the low-income threshold in Community 1, versus 5.8% in Community 2) and multi-ethnicity (with both parents having been born outside Canada in 48.9% of families in Community 1, versus 6.2% of families in Community 2; and both parents speaking only a non-official language at home in 15.4% of families in Community 1, versus 1.6% of families in Community 2). Although Triple P has been proven to be effective across various socio-economic groups, and with culturally and linguistically diverse parents (Eyberg et al. 2008; Nowak and Heinrichs 2008), it is possible that these factors had an influence on the providers’ usage of Triple P with families. Indeed, parents from culturally diverse backgrounds are less likely than other families to access parenting programs (Cunningham et al. 2000; Sawrikar and Katz 2008), despite the fact that culturally diverse children can be at greater risk for developing behavioral problems (McCabe et al. 2005; Willgerodt and Thompson 2006). Parents from Community 1 might have been more difficult to reach and engage in Triple P, which could have contributed to the differences observed in terms of the amount of usage.

Another explanation for the association between the community and usage relates to the implementation process within each community. Case history reports of the implementation process (Delawarde-Saïas et al. 2018; Gagné et al. 2017) suggested that the two communities differed in terms of inter-organizational links and peer support structures. These reports showed that, although both communities experienced episodes of doubt, uncertainty and readjustment, the establishment of a relatively stable implementation committee in Community 2 appears to have fostered the creation of inter-organizational links and strengthened the partnership, helping to reduce initial concerns regarding the implementation of Triple P. Also, Community 2 quickly set up a peer support structure, while in Community 1, this was only done towards the end of the demonstration project period. It is possible that these inter-organizational links and peer support structure promoted the providers’ use of Triple P. This would be consistent with the theoretical literature suggesting that, in the case of a community partnership, broader social and contextual factors such as linkages and connections among the organizations involved, commitment, and the time and effort invested by the partners are essential elements in supporting a quality implementation process and ensuring sustainability of the EBP (Mendel et al. 2008).

Interestingly, the number of years of experience, attitudes toward EBPs, perceived training needs, perceived adequacy of the organization’s physical environment, and perceived organizational facilitators and barriers to implementation were not significant predictors of usage versus non-usage or the amount of usage. One hypothesis that could explain this result is that these attitudes and perceptions may have changed after the training and during the course of the implementation. As highlighted in the case history reports (Delawarde-Saïas et al. 2018; Gagné et al. 2017), providers who were initially skeptical were able to familiarize themselves with the EBP content during training, and stated afterwards that they were reassured by the values advocated by the EBP.

Finally, although model fits for both regressions were good, some variance in usage and amount of usage was unaccounted for by the models. This suggests that while some of the hypothesized relationships between the constructs were confirmed, other factors that were not captured by the measures used in this study may have impacted the service providers’ use of Triple P. This finding is not surprising; indeed, while the providers had some freedom in choosing whether or not to use Triple P with the families they worked with, the presence of factors out of their control that may have influenced usage cannot be ignored. These factors include, but are not limited to: organizational climate, support received in implementing Triple P, requirements from the employer, number of EBPs used in this organization, characteristics of the workload, type and number of referrals received by providers, and characteristics and preferences of the parents consulting in this organization. Such other potential influences on usage deserve careful investigation. Although some of these factors are more difficult to conceptualize and assess, future studies would benefit from examining both provider-level and broader contextual variables as determinants of the use of an EBP.

Taken together, these results indicate that what counts when it comes to implementing and disseminating a new EBP is not so much selecting an experienced, optimistic, and motivated workforce. The factors influencing providers’ use of a new EBP appear to be mainly under the control of the organization (e.g., selection of trained providers, amount of training and support provided), suggesting that these are the factors that can make a difference in the implementation process. Organizations should ensure that providers receive adequate training prior to the implementation, that they have continuous access to supervision and peer support during the implementation, and that measures are taken to enable them to easily incorporate the EBP into their current practice (Sanders et al. 2009; Seng et al. 2006).

Regarding the measure of usage, the results confirmed the heterogeneity of the providers’ practice (use of different levels and modalities of intervention). This heterogeneity should be expected when implementing a multi-level EBPs such as Triple P, and considered when assessing usage. The frequency distribution of the MUI suggested that usage should not be measured merely as a dichotomous variable. It supported the importance of using an integrative and multidimensional measure of the amount of usage to ensure an accurate and exhaustive assessment of the extent of the dissemination. Due to the complexity of assessing the use of multi-level EBPs such as Triple P, the creation of a multidimensional index is particularly relevant in these cases. However, studies examining single-level EBPs or interventions would also benefit from incorporating a similar index to capture multiple aspects of delivery and achieve a more comprehensive picture of implementation processes.

Some limitations of the present study must be underlined. First, data collection relied exclusively on provider self-reports, which do not provide objective data on the implementation process. Although the usage data were monitored by the research team, providers may have tended to overestimate or underestimate certain indicators of the amount of usage. For example, when completing the monitoring instruments, they may have rounded up the total duration of the interventions conducted. They may also have omitted or forgotten to complete monitoring instruments for some interventions, especially individual interventions, as the research team was not systematically aware of all such interventions carried out. Moreover, while the present study confirmed the importance of the community in predicting usage, another limitation is that it did not include measures relating to the implementation context that would have made it possible to distinguish between the characteristics of the two communities. Therefore, the community-level factors that could have impacted the amount of usage remain unclear.

Despite these limitations, the present study provides a holistic conceptualization of the extent to which various characteristics of the providers and implementation context are important factors when it comes to predicting the use of an EBP. The MUI constitutes another important contribution of the present study, allowing for a more accurate and exhaustive assessment of the extent of the dissemination of a multi-level EBP. Also, the indicators of the amount of usage were assessed using prospective measures, avoiding the risk of recall bias. The fact that the variables related to the providers’ usage of an EBP were examined among a multisectoral sample of providers is another strength of this study. Future studies examining the implementation of other EBPs could build on the experience and outcomes of the present study, ultimately leading to more effective implementation processes and assessment strategies. These studies would benefit from including measures of specific characteristics of the implementation context in order to identify and better understand the community-level factors promoting higher usage of EBPs.
